# High transmission risk in HIV-1 molecular transmission network among MSM is related to unsafe sexual behavior and adverse childhood experiences: a case-control study

**DOI:** 10.3389/fpubh.2025.1678216

**Published:** 2025-12-18

**Authors:** Zijia Lin, Ruixuan Wei, Yefei Luo, Mengjun Li, Yan Zhuang, Shuqing Yin, Liyun Jiang, Qingmei Li, Hao Wu, Peng Xiong, Zhigang Han

**Affiliations:** 1 Department of Public Health and Preventive Medicine, School of Medicine, Jinan University, Guangzhou, China; 2 Guangzhou Center for Disease Control and Prevention, Guangzhou, China; 3 Dazhou Key Laboratory for Precision Cancer Therapy, Dazhou Key Laboratory for Artificial Intelligence and Medical Imaging, Department of Clinical Research Center, Sichuan Clinical Research Center for Medical Imaging, Dazhou Central Hospital, Dazhou, China; 4 School of Public Health, Southern Medical University, Guangzhou, China; 5 Laboratory of Neuromanagement, Guangdong Key Laboratory of Philosophy and Social Sciences, Jinan University, Guangzhou, China

**Keywords:** phylogenetics, MSM, adverse childhood experiences, HIV-1 infections, sexual behavior

## Abstract

**Background:**

Molecular transmission networks enable successful identification of core transmitters compared to traditional epidemiological surveillance; however, behavioral characteristics and psychological drivers of these spreaders remain poorly characterized. Adverse childhood experiences (ACEs) are significantly more common among HIV-positive individuals than in the general population; yet empirical evidence showing that ACEs increase transmission risk among men who have sex with men (MSM) remains limited. This study investigates transmission risk in molecular network associations with sexual behaviors and adverse childhood experiences among MSM.

**Methods:**

This study was a Case–Control Study based on molecular networks. A molecular transmission network was constructed using HIV-1 pol sequences from 1,691 newly diagnosed MSM in Guangzhou (2018–2020). Cases were defined as individuals with a network degree ≥3 (high transmission risk group) and controls were defined as those with a degree <3 (low transmission risk group), matched 1:1 by age (±5 years). Data on sexual behavior and adverse childhood experiences were collected via electronic questionnaires from 2023 to 2024. Logistic regression was used to analyze associations between these factors and transmission risk.

**Results:**

Among 1,691 participants, 40.57% were included in the molecular network, comprising 238 high-risk and 448 low-risk individuals. After matching, 119 pairs were analyzed. High-risk MSM exhibited significantly elevated transmission risks associated with sexually transmitted infections (aOR = 2.947, 95% CI: 1.084–8.008); versatile sexual role (aOR = 2.856, 95% CI: 1.323–6.165); infrequent sexual activity (monthly: aOR = 6.487, 95% CI: 1.594–26.407; ≤quarterly: aOR = 6.708, 95% CI: 1.668–26.984); no stable partner or multiple stable partners (aOR = 2.516, 95% CI: 1.231–5.140); childhood sexual abuse (aOR = 2.791, 95% CI: 1.268–6.146); physical neglect (aOR = 2.386, 95% CI: 1.087–5.238).

**Conclusion:**

Unsafe sexual behavior and ACEs significantly increased the likelihood of becoming a core transmitter within MSM networks. Integrating screening for these factors into prevention programs could optimize early identification of high-transmission-risk MSM and enhance precision interventions.

## 1 Introduction

There were approximately 40.8 million people living with HIV at the end of 2024 with 1.3 million people becoming newly infected with HIV in 2024 globally. An estimated 0.7% of adults aged 15–49 years worldwide are living with HIV, although the burden of the epidemic continues to vary considerably between countries and regions. The low-and-middle-income-countries and low-income-countries remain most severely affected, such as African region, with nearly 1 in every 30 adults (3.1%) living with HIV and accounting for more than two-thirds of the people living with HIV worldwide (1). In China, although comprehensive strategies such as the “Four Frees and One Care” policy have successfully maintained an overall low-level epidemic (2), the men who have sex with men (MSM) persists as a core driver of HIV transmission due to prevalent high-risk behaviors (3, 4). From 2006 to 2022, the proportion of newly reported HIV cases attributed to MSM rose from 2.5 to 25.6% (2). The persistently high prevalence rate among MSM (5) indicated that MSM remained a critical challenge for current prevention efforts, and conventional strategies are face bottlenecks in effectively interrupting transmission chains within this demographic.

Molecular network analysis offers a novel perspective for addressing prevention challenge mentioned above, by examining genetic relatedness among HIV strains to construct molecular transmission networks. It enables rapid and accurate identification of transmission clusters and pinpoints high-transmission-risk individuals occupying key network nodes (6–8). This technology effectively complements traditional epidemiological investigation. Research indicated that core transmitters, constituting only about 12% of the network, but accounted for over 22% of new infections (9). Therefore, in-depth investigation into the behavioral characteristics and related influencing factors of high-transmission-risk individuals is crucial for achieving efficient and targeted HIV prevention and control. However, existing research predominantly focuses on the network structure itself, with significantly insufficient exploration of the specific behavioral traits of core transmitters and their underlying psychosocial drivers, particularly sexual behavior patterns (3, 10–12). Consequently, the epidemiological characteristics of HIV-positive individuals within networks—especially factors related to transmission risk—have not been well-studied.

Concurrently, substantial evidence demonstrated that the prevalence of adverse childhood experiences (ACEs) is significantly higher among people living with HIV (PLWH) compared to the general population (13). Furthermore, MSM were more likely to report ACEs than men in the general population (14). ACEs have been confirmed to exert profound negative effects on individuals’ mental health, emotional regulation capacity, substance use, and sexual behavior patterns, significantly increasing the risk of engaging in unsafe sexual practices (15–17). Although ACEs’ role in individual risk of HIV acquisition is recognized, their influence on HIV transmission risk, particularly core transmitter risk among MSM lacks empirical investigation.

To address this gap, this study integrated molecular epidemiology and social-behavioral research by constructing molecular transmission networks to quantify individual transmission risk, thereby identifying high/low-transmission-risk individuals and locating core transmitters within the molecular network. Employing a matched case–control study design, we retrospectively collected data on sexual behavior characteristics and ACEs among MSM core transmitters to investigate their relationship with transmission risk. This approach provides a scientific basis for developing targeted intervention strategies that integrate biomedical and psychosocial risk factors.

## 2 Subjects and methods

### 2.1 PCR amplification and molecular network construction

A total of 1,691 newly diagnosed MSM living with HIV-1 in Guangzhou from 2018 to 2020 were enrolled in this study. Inclusion criteria were: (1) residence in Guangzhou (excluding foreign nationals); (2) transmission route attributed to male–male sexual behavior; (3) no prior antiretroviral therapy (ART) at diagnosis. Exclusion criteria included: (1) failure in HIV RNA extraction, amplification, or sequencing; (2) insufficient serum samples for testing.

HIV RNA was extracted from pre-ART plasma samples. The pol region (HXB2 genome positions 2,244–3,821) was amplified via RT-PCR, followed by purification and sequencing. Sequences were edited and aligned using Chromas Pro 2.1.3 and MEGA 11.0, respectively, with reference sequences from the HIV-1 subtype database.^1^ Molecular transmission networks were built using HIV-TRACE with a genetic distance threshold of 1.5% (18–20), employing the TN93 substitution model for pairwise comparisons. Network metrics (degree, betweenness centrality, average shortest path length, closeness centrality, clustering coefficient) were analyzed using Cytoscape 3.7.0.

### 2.2 Data collection

Based on the results of molecular transmission network analysis, nodes with degree values in the upper quartile (degree ≥3) were classified as high-risk transmitters, while those with degree <3 were defined as low-risk transmitters (9, 21, 22). High-risk transmitters (case group) and low-risk transmitters (control group) were matched 1:1 by age (±5 years). Inclusion criteria required: (1) residency in Guangzhou during the study period; (2) willingness to complete questionnaires; (3) age ≥18 years. Exclusions included: (1) non-residency in Guangzhou; (2) severe mental illness, communication barriers, or refusal to participate. Demographic data (age, residence, marital status, education, reporting region, transmission route) were retrieved from the China CDC HIV/AIDS Comprehensive Prevention and Control Information System. Sexual behavior characteristics and ACEs were assessed via electronic questionnaires administered between July 2023 and January 2024. This assessment was conducted at the infectious disease clinics of two designated HIV treatment hospitals in Guangzhou: The Eighth Affiliated Hospital of Guangzhou Medical University and Nanfang Hospital of Southern Medical University. Participants from other treatment sites were contacted by telephone and invited to complete the survey on-site. The survey included: General demographics, sexual behavior characteristics and adverse childhood experiences. It was designed with logic checks to improve data reliability. The survey steps included the research purpose explanation, informed consent signing and on-site verification. Participants were reimbursed with a time-loss subsidy for their involvement.

### 2.3 Key variable

#### 2.3.1 Sexually transmitted infections (STIs)

Diagnosis of any STI (e.g., syphilis, genital warts, herpes, gonorrhea, chlamydia) prior to HIV infection.

#### 2.3.2 Substance abuse

Lifetime use of psychoactive substances, including Rush, 0-number capsules (poppers), Viagra, heroin/morphine/opium, cannabis, ketamine/methamphetamine/Magu/Yaotouwan/benzodiazepines, or psychiatric medications.

#### 2.3.3 Adverse childhood experiences (ACEs)

ACEs was assessed using the validated Chinese Childhood trauma questionnaire-short form (CTQ-SF) (23, 24), covering five domains: emotional abuse (score ≥13), physical abuse (≥10), sexual abuse (≥8), emotional neglect (≥15), and physical neglect (≥10). Participants not meeting thresholds in any domain were classified as “no ACEs.” Internal consistency was excellent (Cronbach’s *α* = 0.85).

### 2.4 Statistical analysis

Data were analyzed using R software (version 4.3.4). Categorical variables were summarized using frequency distributions. Demographic differences between transmission risk groups were compared via chi-square (*χ*^2^) tests. Factors associated with HIV transmission risk were analyzed using univariate and multivariate conditional logistic regression models, with high/low transmitters as the dependent variable. Variables with *p* < 0.1 in univariate analysis or requiring adjustment were included in the multivariate model. Statistical significance was set at *p* < 0.05. Multicollinearity among variables was assessed using variance inflation factors (VIF).

## 3 Results

### 3.1 Demographic characteristics of MSM living with HIV-1 in Guangzhou (2018–2020)

A total of 1,691 newly diagnosed MSM living with HIV-1 were included. Over half (51.9%) were 21–30 years old. The majority were Han Chinese (94.6%), unmarried (79.1%), employed (67.6%), and held household registration in Guangdong Province (51.8%). Nearly half (47.3%) had attained college-level education or higher. Details are shown in Supplementary Table 1.

### 3.2 Molecular transmission network analysis

**Figure 1:**
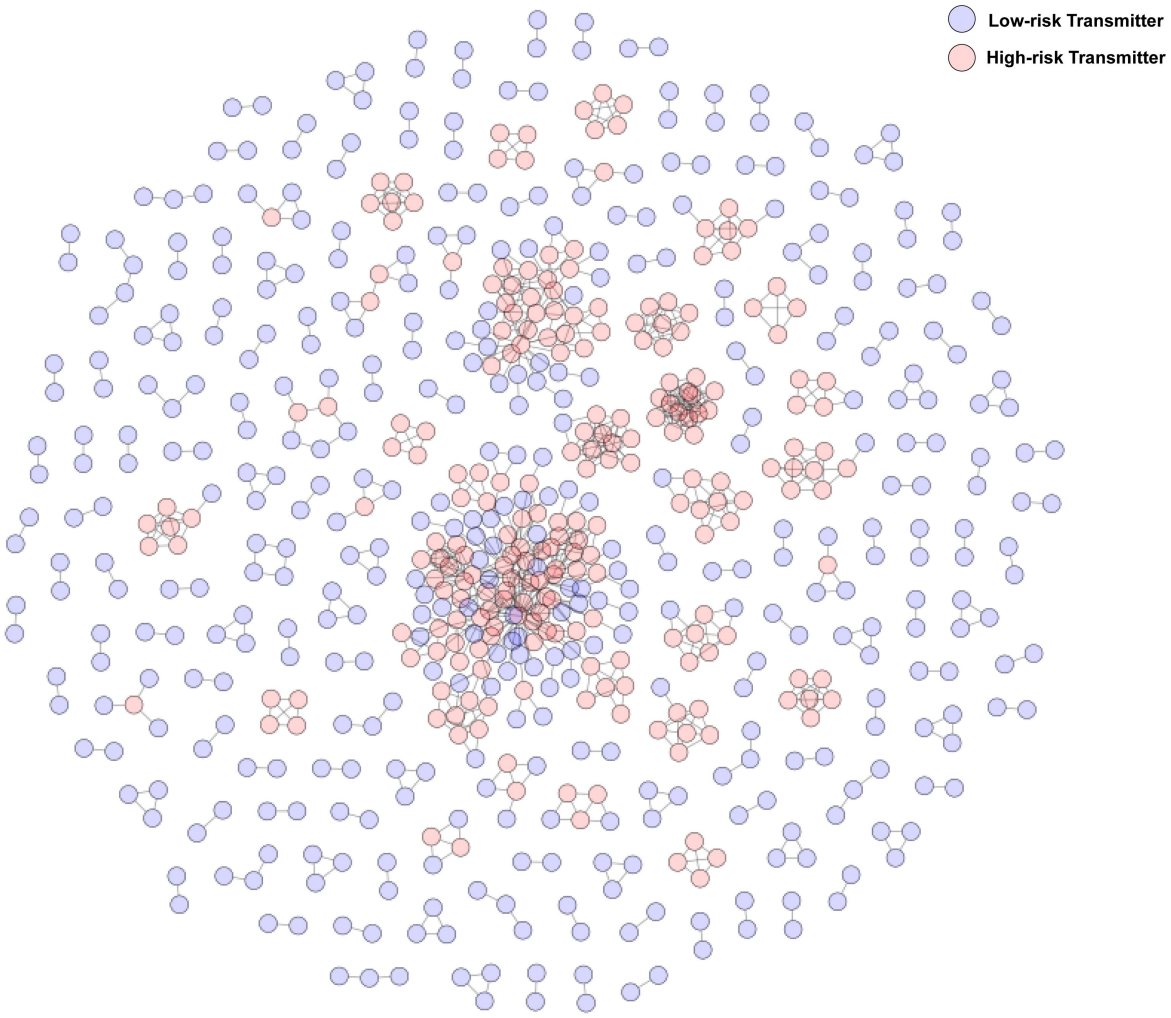
Molecular transmission network of HIV-1 among MSM in Guangzhou (2018–2020).

At a genetic distance threshold of 1.5, 40.57% (686/1,691) of cases were integrated into the molecular network and formed 178 clusters (size range: 2–135). Among these, 238 (34.7%) were classified as high-risk transmitters and 448 (65.3%) as low-risk transmitters, e.g., (Figure 1).

As shown in Table 1, high-risk transmitters exhibited significantly higher betweenness centrality (0.01 vs. 0.00, *p* < 0.001) and clustering coefficient (0.69 vs. 0.00, *p* < 0.001) compared to low-risk transmitters. Conversely, high-risk transmitters had longer average shortest path lengths (1.52 vs. 1.00, *p* < 0.001) and lower closeness centrality (0.66 vs. 1.00, *p* < 0.001).

**Table 1:** Centrality analysis of the HIV-1 molecular network among MSM in Guangzhou, 2018–2020 (*N* = 686).

Centrality metric (M(IQR))	High-risk transmitter (*n* = 238)	Low-risk transmitter (*n* = 448)	*Z*	*p*
Betweenness centrality	0.01 (0.07)	0.00 (0.00)	−16.47	<0.001
Closeness centrality	0.66 (0.58)	1.00 (0.33)	−8.96	<0.001
Average shortest path length	1.52 (1.84)	1.00 (0.50)	−8.96	<0.001
Clustering coefficient	0.69 (0.61)	0.00 (0.00)	−12.96	<0.001

### 3.3 Baseline characteristics by transmission risk group

Among 686 network-linked MSM, low-risk transmitters had higher educational attainment (*p* < 0.05) than high-risk transmitters. No statistically significant differences were observed in age, ethnicity, marital status, occupation, or household registration between the two groups (Table 2).

**Table 2:** Demographic characteristics of high- and low-risk transmitters.

Demographic characteristic	High-risk transmitter (*N* = 238), *n* (%)	Low-risk transmitter (*N* = 448), *n* (%)	*χ^2^*	*P*
Age (years)			8.28	0.082
≤20	40(16.8)	55(12.3)		
21–30	116(48.7)	255(56.9)		
31–40	43(18.1)	88(19.6)		
41–50	30(12.6)	35(7.8)		
>50	9(3.8)	15(3.3)		
Ethnicity			3.13	0.077
Han	223(93.7)	434(96.9)		
Other	15(6.3)	14(3.1)		
Marital status			2.42	0.489
Unmarried	195(81.9)	375(83.7)		
Married	28(11.8)	46(10.3)		
Divorced/widowed	13 (5.5)	18 (4.0)		
Missing data	2 (0.8)	9 (2.0)		
Education level			9.12	0.028
Junior high or below	65 (27.3)	91 (20.3)		
High school/technical	64 (26.9)	112 (25.0)		
College or above	108 (45.4)	233 (52.0)		
Missing data	1 (0.4)	12 (2.7)		
Occupation			5.42	0.247
Employed	156 (65.5)	299 (66.7)		
Unemployed	30 (12.6)	46 (10.3)		
Freelancer	4 (1.7)	18 (4.0)		
Student	29 (12.2)	41 (9.2)		
Missing data	19 (8.0)	44 (9.8)		
Household registration			1.22	0.544
Guangzhou city	37 (15.5)	58 (12.9)		
Other cities in Guangdong	88 (37.0)	161 (35.9)		
Other provinces	113 (47.5)	229 (51.1)		

### 3.4 Analysis of factors influencing HIV transmission risk

As shown in Table 3, 72.6% of MSM living with HIV within the transmission network had experienced at least one ACE. Specifically, among MSM reporting sexual abuse history (*n* = 53, 22.2%), 62.26% were in the high-risk group; for physical neglect (*n* = 152, 63.87%), 55.26% were high-risk transmission. Similarly, 65.91% of STI cases and 65.08% of those reporting insertive/versatile sexual roles belonged to the high-risk group. Additionally, in terms of seeking sexual partners, 83.6% of the individuals primarily used online channels. Among study participants who primarily sought sexual partners online, high-risk transmitters accounted for 62.26%, but high-risk transmitters sought sexual partners online less frequently than low-risk transmitters.

**Table 3:** Factors associated with HIV transmission risk among MSM.

Variable	Low-risk (*N* = 119), *n* (%)	High-risk (*N* = 119), *n* (%)	Unadjusted OR (95%CI)	*P*	Adjusted OR (95%CI)	*P*
BMI						
Normal	81 (54.36%)	68 (45.64%)	Ref			
Underweight	22 (50%)	22 (50%)	1.09 (0.572, 2.077)	0.794		
Overweight	11 (33.33%)	22 (66.67%)	2.575 (1.103, 6.012)	0.029		
Obesity	5 (41.67%)	7 (58.33%)	1.8 (0.545, 5.943)	0.335		
Ethnicity						
Han	115 (49.78%)	116 (50.22%)	Ref		Ref	
Other	4 (57.14%)	3 (42.86%)	0.75 (0.168, 3.351)	0.706	0.376 (0.064, 2.203)	0.278
Education level						
Junior high or below	16 (43.24%)	21 (56.76%)	Ref		Ref	
High school/vocational	29 (46.77%)	33 (53.23%)	0.774 (0.325, 1.847)	0.564	0.63 (0.202, 1.97)	0.427
College or above	74 (53.24%)	65 (46.76%)	0.602 (0.261, 1.387)	0.233	0.515 (0.161, 1.65)	0.264
Occupation						
Full-time	95 (49.74%)	96 (50.26%)	Ref			
Part-time/unemployed/self-employed/retired	22 (53.66%)	19 (46.34%)	0.85 (0.445, 1.623)	0.622		
Student	2 (33.33%)	4 (66.67%)	2 (0.366, 10.919)	0.423		
Income (RMB)						
≤3,000	15 (46.88%)	17 (53.12%)	Ref		Ref	
3,001–5,000	35 (50%)	35 (50%)	0.922 (0.412, 2.065)	0.844	0.847 (0.307, 2.333)	0.748
5,001–7,000	26 (45.61%)	31 (54.39%)	1.097 (0.467, 2.576)	0.831	0.596 (0.201, 1.771)	0.352
>7,000	43 (54.43%)	36 (45.57%)	0.702 (0.305, 1.615)	0.405	0.538 (0.187, 1.547)	0.25
Marital status						
Unmarried	100 (49.5%)	102 (50.5%)	Ref		Ref	
Married	11 (50%)	11 (50%)	0.9 (0.273, 2.967)	0.862	0.77 (0.18, 3.298)	0.725
Divorced/widowed	8 (57.14%)	6 (42.86%)	0.728 (0.239, 2.215)	0.576	0.567 (0.128, 2.501)	0.454
Gender identity						
Male	109 (51.42%)	103 (48.58%)	Ref			
Female	3 (50%)	3 (50%)	1.058 (0.211, 5.319)	0.945		
Both	5 (41.67%)	7 (58.33%)	1.407 (0.443, 4.468)	0.563		
Other	2 (25%)	6 (75%)	3 (0.606, 14.864)	0.178		
Sexual orientation						
Homosexual	83 (50.92%)	80 (49.08%)	Ref			
Heterosexual	2 (25%)	6 (75%)	4.414 (0.796, 24.467)	0.089		
Bisexual	26 (59.09%)	18 (40.91%)	1.444 (0.714, 2.922)	0.307		
Other	8 (34.78%)	15 (65.22%)	2.916 (0.967, 8.786)	0.057		
Primary way of seeking sexual partners						
Offline	23 (58.97%)	16 (41.03%)	Ref		Ref	
Online	96 (48.24%)	103 (51.76%)	1.636 (0.773, 3.465)	0.198	1.983 (0.743, 5.291)	0.171
Frequency of sexual behavior						
About once a week	15 (68.18%)	7 (31.82%)	Ref		Ref	
About once a month	36 (46.75%)	41 (53.25%)	2.391 (0.875, 6.531)	0.089	6.487 (1.594, 26.407)	0.009
Once every 3 months or less	68 (48.92%)	71 (51.08%)	2.278 (0.835, 6.211)	0.108	6.708 (1.668, 26.984)	0.007
Sexual role						
Receptive	97 (55.43%)	78 (44.57%)	Ref		Ref	
Insertive/versatile	22 (34.92%)	41 (65.08%)	2.267 (1.235, 4.161)	0.008	2.856 (1.323, 6.165)	0.008
Group sex						
Yes	10 (41.67%)	14 (58.33%)	Ref			
No	109 (50.93%)	105 (49.07%)	0.667 (0.273, 1.631)	0.374		
Condom use frequency						
Never	1 (50%)	1 (50%)	Ref			
Sometimes	83 (50.3%)	82 (49.7%)	0.978 (0.06, 15.88)	0.987		
Always	35 (49.3%)	36 (50.7%)	1.023 (0.063, 16.61)	0.987		
Number of regular sexual partners						
Single fixed partner	73 (45.91%)	86 (54.09%)	Ref		Ref	
No/multiple partners	46 (58.23%)	33 (41.77%)	1.684 (0.955, 2.971)	0.072	2.516 (1.231, 5.14)	0.011
Casual sexual partners						
No	46 (50%)	46 (50%)	Ref		Ref	
Yes	73 (50%)	73 (50%)	1 (0.592, 1.69)	1	0.744 (0.379, 1.46)	0.391
Sexually transmitted infections (STIs)						
No	104 (53.61%)	90 (46.39%)	Ref		Ref	
Yes	15 (34.09%)	29 (65.91%)	3 (1.275, 7.057)	0.012	2.947 (1.084, 8.008)	0.034
Commercial sexual behavior						
No	6 (66.67%)	3 (33.33%)	Ref			
Yes	113 (49.34%)	116 (50.66%)	2 (0.5, 7.997)	0.327		
Drug abuse						
No	79 (50%)	79 (50%)	Ref			
Yes	40 (50%)	40 (50%)	1 (0.568, 1.761)	1		
ACEs						
No	40 (61.54%)	25 (38.46%)	Ref			
Yes	79 (45.66%)	94 (54.34%)	1.937 (1.06, 3.542)	0.032		
Emotional abuse						
No	103 (50%)	103 (50%)	Ref			
Yes	16 (50%)	16 (50%)	1 (0.416, 2.403)	1		
Physical abuse						
No	104 (50.49%)	102 (49.51%)	Ref			
Yes	15 (46.88%)	17 (53.12%)	1.167 (0.54, 2.522)	0.695		
Sexual abuse						
No	99 (53.51%)	86 (46.49%)	Ref		Ref	
Yes	20 (37.74%)	33 (62.26%)	1.765 (0.973, 3.199)	0.061	2.791 (1.268, 6.146)	0.011
Emotional neglect						
No	62 (46.62%)	71 (53.38%)	Ref			
Yes	57 (54.29%)	48 (45.71%)	0.719 (0.421, 1.228)	0.227		
Physical neglect						
No	51 (59.3%)	35 (40.7%)	Ref		Ref	
Yes	68 (44.74%)	84 (55.26%)	2 (1.097, 3.645)	0.024	2.386 (1.087, 5.238)	0.03

Univariate regression analysis revealed that MSM with a history of ACEs (OR = 1.937, 95% CI: 1.060–3.542) had significantly higher HIV transmission risks. Additional factors associated with elevated transmission risk included: overweight (OR = 2.575, 95% CI: 1.103–6.012), insertive or versatile sexual role (OR = 2.267, 95% CI: 1.235–4.161), sexually transmitted infections (STIs) (OR = 3.000, 95% CI: 1.275–7.057), childhood sexual abuse (OR = 1.765, 95% CI: 0.973–3.199), and physical neglect (OR = 2.000, 95% CI: 1.097–3.645). All variables passed collinearity checks, with tolerance values >0.8 and variance inflation factors (VIF) <2, indicating no significant multicollinearity. Multivariate regression (Table 3), adjusted for ethnicity, education level, income, marital status, Primary Way of Seeking Sexual Partners, and casual sexual partners, identified the following independent predictors of higher transmission risk: STIs (aOR = 2.947, 95% CI: 1.084–8.008), Versatile sexual role (aOR = 2.856, 95% CI: 1.323–6.165), Childhood sexual abuse (aOR = 2.791, 95% CI: 1.268–6.146), Childhood physical neglect (aOR = 2.386, 95% CI: 1.087–5.238), Infrequent sexual activity: Monthly (aOR = 6.487, 95% CI: 1.594–26.407), Quarterly or less (aOR = 6.708, 95% CI: 1.668–26.984), No stable partner or multiple stable partners (aOR = 2.516, 95% CI: 1.231–5.140).

## 4 Discussion

This study quantified HIV transmission risk among MSM by constructing a molecular transmission network model, the result indicated that this group occupied more central nodal positions and demonstrated a greater tendency to form tight local transmission clusters with surrounding nodes (25, 26). Notably, the high-transmission-risk group also held influential network positions with the potential for broad dissemination, but their transmission paths may not be the most straightforward, and they possess the potential for cross-group diffusion (27). Collectively, these network topological characteristics underscore the heightened transmission risk associated with this group. Building upon this molecular epidemiological evidence, this study further analysis revealed that these core nodes in the transmission network (i.e., high-risk transmitters) were significantly associated with exposure to ACEs and specific sexual behavioral traits, including versatile sexual role behavior and having multiple sexual partners.

Existing studies revealed the high prevalence of childhood sexual abuse among MSM (28, 29). This study found that 72.6% of MSM living with HIV had experienced at least one ACE, significantly higher than the 61.6% ACE prevalence reported in previous studies among the general MSM population (30). Further analysis revealed that the high-transmission-risk group exhibited a significantly greater prevalence of ACEs (OR = 1.937, 95% CI: 1.060–3.542) compared to the low-risk group. Notably, childhood sexual abuse (aOR = 2.791, 95% CI: 1.268–6.146) and physical neglect (aOR = 2.386, 95% CI: 1.087–5.238) were particularly prominent among the exposure subtypes. These findings suggest that MSM living with HIV and having a history of ACEs may have an elevated potential for transmission risk. This finding aligns with established theories linking childhood sexual abuse to high-risk sexual behaviors (29, 31–33). Supporting evidence from O’Leary et al. indicated that ACEs-exposed MSM living with HIV are more prone to engage in condomless sex with serodiscordant or status-unknown partners (17). Neurobiological mechanisms suggested that childhood maltreatment may disrupt hypothalamic–pituitary–adrenal axis function and alter parahippocampal-limbic circuit activity, promoting emotional dysregulation and impulsivity (34, 35). These effects contribute to impaired cognitive and socioemotional functioning in adulthood (36), thereby increasing vulnerability to risk-enhancing factors in sexual partner selection and behavioral decision-making. Furthermore, sexual abuse survivors often face barriers in acquiring HIV prevention knowledge, manifesting as weakened self-protection awareness and an increased likelihood of neglecting safety measures during sexual encounters (37), while ACEs may reduce adherence to HIV treatment and prevention protocols among seropositive individuals, thereby amplifying transmission risks (38). Notably, ACE survivors frequently co-occur with mental health comorbidities such as anxiety and depression (38, 39), and these psychological conditions may further exacerbate risky sexual practices (40).

This study, consistent with prior research (41, 42), demonstrated that STIs (aOR = 2.947, 95% CI: 1.084–8.008) significantly increase HIV transmission risks among MSM living with HIV. Most STIs share overlapping transmission routes and risk factors with HIV/AIDS, exhibiting synergistic effects at both biological and behavioral levels (43, 44). STI-induced genital inflammation and ulcerations compromised mucosal barrier integrity, thereby elevating the probability of HIV viral entry (45). In some regions, high STI prevalence was closely associated with HIV transmission dynamics. Consequently, enhancing STI prevention and treatment efforts is a critical measure for reducing HIV transmission risks (45–48).

Sexual role is a defining characteristic of MSM populations, with differential HIV transmission risks across roles (49). Consistent with previous observations, our findings indicated that insertive or versatile role (aOR = 2.856, 95% CI: 1.323–6.165) in MSM with HIV is associated with significantly heightened HIV transmission potential (50). However, receptive partners have long been recognized as facing elevated HIV acquisition risks during anal intercourse due to the vulnerability of rectal mucosa (51). Although insertive partners face lower physiological susceptibility to mucosal trauma, their role as primary decision-makers in condom use may paradoxically amplify risks (52). Specifically, insertive partners often prioritize heightened sexual stimulation over protection, leading to intentional condom avoidance (53). Furthermore, versatile MSM frequently switch roles during sexual encounters but exhibit inconsistent condom use during these transitions (51). This pattern of behavior can facilitate the dissemination of HIV across different sexual networks by bridging distinct risk clusters through their dual-role interactions (51, 54).

This study found that HIV-positive MSM individuals without stable partners or with multiple concurrent partnerships (aOR = 2.516, 95% CI: 1.231–5.140) face higher risks of HIV transmission compared to those with only one steady sexual partner. This consisted with other studies that steady partnerships play a positive role in reducing the number of sexual partners (55). Paradoxically, increased numbers of stable partners correlated with higher rates of condomless anal intercourse (56). This association likely stems from misplaced trust in stable partner serostatus disclosure, which individuals may forgo protection, thus heightening transmission risks (53). It is noteworthy that no significant difference in condom use frequency was observed between the two groups, this may be related to the widespread improper condom use within this population. Therefore, consistently and correctly using protection during sexual activities remains crucial for effectively preventing HIV infection and transmission.

While online dating has become a primary platform for MSM to seek partners, and prior studies link dating app use to high-risk behaviors and STI transmission (57, 58), this study observed paradoxically lower transmission risk among frequent online daters (monthly: aOR = 6.487, 95% CI: 1.594–26.407; ≤quarterly: aOR = 6.708, 95% CI: 1.668–26.984). Potential explanations include: (1) Undifferentiated categorization of partner types (e.g., frequent interactions may involve safer, regular partners); (2) Underreporting of high-risk behaviors due to social desirability bias among casual partner seekers; (3) MSM populations demonstrate inherent vigilance toward online-acquired partners, resulting in higher condom utilization rates (59–61); (4) Low-frequency online users might exhibit compensatory risk-taking through offline sexual encounters. Future research should disentangle partner typologies, relationship stability, and online-offline behavioral interplay to refine HIV risk assessment models.

This study has several methodological limitations. First, the completeness of the molecular transmission network was inherently limited because blood samples could not be collected from all HIV-infected individuals in the target population, and the availability of HIV RNA sequences depended on clinical sampling protocols. This may led to the underestimation of network connectivity and missed some transmission links, potentially affecting the identification of all core transmitters. Second, self-reported behavioral data may be subject to recall bias and intentional non-disclosure, particularly given the sensitive nature of questions related to ACEs and sexual behaviors. While anonymized surveys were employed to mitigate social desirability bias, the accuracy of responses could not be fully validated. Furthermore, due to the cross-sectional design of the study, the temporal relationship and causality between adverse childhood experiences, sexual behaviors, and transmission risk remain unclear. Finally, as the findings are derived from a sample of MSM in Guangzhou, China, they may not be readily generalized to other regions or cultural settings. Therefore, caution is necessary when extrapolating these results to other populations.

## 5 Conclusion

To effectively curb HIV transmission, we recommend: (1) Integrating ACEs screening into HIV control programs to enable early identification of high-transmission-risk MSM and delivery of trauma-informed interventions; (2) Proactively scaling up antiviral treatment and partner-engagement strategies targeting core risk groups—insertive/versatile MSM and those without/multiple stable partners; (3) Establishing dynamic sexual network surveillance to decode the paradoxical online-risk association, replacing one-size-fits-all approaches. Precision identification of high-transmission-risk populations will optimize resource allocation for evidence-based interventions.

## Data Availability

The datasets presented in this article are not readily available because our institution has a data access and sharing policy that outlines the procedures for data storage, access, and eventual sharing or archiving of data. We are bound by these policies. Requests to access the datasets should be directed to zhiganghan616@163.com.

## Footnotes

1
www.hiv.lanl.gov
