# Supplementary material for: High transmission risk in HIV-1 molecular transmission network among MSM is related to unsafe sexual behavior and adverse childhood experiences: a case-control study
Source: Front Public Health. 2025 Dec 18;13:1678216. doi: 10.3389/fpubh.2025.1678216 (PMC12756416; doi:10.3389/fpubh.2025.1678216)
Supplement: Supplementary file 1 [file Table_1.DOCX]

**Supplemental** **Table 1 Demographic Characteristics of MSM Living with HIV-1 in Guangzhou (2018–2020)（N=1691）**

| **Variable** | **Number** | **Proportion (%)** |
| --- | --- | --- |
| **Age group(years)** |  |  |
| ≤20 | 164 | 9.7 |
| 21-30 | 879 | 52.0 |
| 31-40 | 376 | 22.2 |
| 41-50 | 195 | 11.5 |
| >50 | 77 | 4.6 |
| **Ethnicity** |  |  |
| Han | 1600 | 94.6 |
| Other | 91 | 5.4 |
| **Marital status** |  |  |
| Unmarried | 1338 | 79.1 |
| Married | 228 | 13.5 |
| Divorced/Widowed | 93 | 5.5 |
| Missing data | 32 | 1.9 |
| **Education level** |  |  |
| Junior high or below | 420 | 24.8 |
| High school/technical | 444 | 26.3 |
| College or above | 800 | 47.3 |
| Missing data | 27 | 1.6 |
| **Occupation** |  |  |
| Employed | 1143 | 67.6 |
| Unemployed | 201 | 11.9 |
| Freelancer | 54 | 3.2 |
| Student | 130 | 7.7 |
| Retired | 1 | 0.1 |
| Missing data | 162 | 9.6 |
| **Household registration** |  |  |
| Guangzhou city | 286 | 16.9 |
| Other cities in Guangdong | 529 | 31.3 |
| Other provinces | 876 | 51.8 |
